# A lung abscess caused by secondary syphilis – the utility of polymerase chain reaction techniques in transbronchial biopsy: a case report

**DOI:** 10.1186/s12879-019-4236-4

**Published:** 2019-07-09

**Authors:** Shinji Futami, Takayuki Takimoto, Futoshi Nakagami, Shingo Satoh, Masanari Hamaguchi, Muneyoshi Kuroyama, Kotaro Miyake, Shohei Koyama, Kota Iwahori, Haruhiko Hirata, Izumi Nagatomo, Yoshito Takeda, Hiroshi Kida, Atsushi Kumanogoh

**Affiliations:** 1 Department of Respiratory Medicine and Clinical Immunology, Osaka University Graduate School of Medicine, 2-2 Yamada-oka, Suita, Osaka, 565-0871 Japan; 2 Department of General Internal Medicine, Osaka University Hospital, Suita, Osaka, Japan; 3 Department of Internal Medicine, National Hospital Organization, Kinki-Chuo Chest Medical Center, Sakai, Osaka, Japan

**Keywords:** Secondary syphilis, Lung abscess, Polymerase chain reaction, Transbronchial biopsy, Surgical treatment, Case report

## Abstract

**Background:**

In Japan and other countries, the number of patients with syphilis is increasing year by year. Recently, the cases of the pulmonary involvement in patients with secondary syphilis have been reported. However, it is still undetermined how to obtain a desirable specimen for a diagnosis of the pulmonary involvement, and how to treat it if not cured.

**Case presentation:**

A 34-year-old man presented with cough and swelling of the right inguinal nodes. A physical examination revealed erythematous papular rash over the palms, soles and abdomen. A 4 cm mass in the right lower lobe of the lung was detected on computed tomography. He was diagnosed as having secondary syphilis, because he was tested positive for the rapid plasma reagin and *Treponema pallidum* hemagglutination assay. Amoxycillin and probenecid were orally administered for 2 weeks. Subsequently, rash and serological markers were improved, however, the lung mass remained unchanged in size. Transbronchial biopsy (TBB) confirmed the pulmonary involvement of syphilis using polymerase chain reaction techniques (tpp47- and polA-PCR). Furthermore, following surgical resection revealed the lung mass to be an abscess.

**Conclusions:**

To our knowledge, this is the first surgically treated case of a lung abscess caused by syphilis, which was diagnosed by PCR techniques in TBB. This report could propose a useful diagnostic method for the pulmonary involvement of syphilis.

## Background

Syphilis is a sexually transmitted disease caused by infection with *Treponema pallidum*, which is classified into four stages (primary, secondary, latent and tertiary). If the patients with primary syphilis do not receive treatment, the bacterium will spread through their bloodstream, and set the stage for secondary syphilis. Syphilis can cause a wide range of systemic manifestations, such as papular rash, malaise, weight loss, muscle aches, generalized lymphadenopathy and meningitis [1]. In Japan and other countries, the number of patients with syphilis is increasing year by year [2–4]. Recently, several dozen reports showed the pulmonary involvement in patients with secondary syphilis [5–16]; however, it is still undetermined how to obtain a desirable specimen for a diagnosis of the pulmonary involvement, and how to treat it if not cured.

Here, we report a rare case of a lung abscess caused by secondary syphilis, that was definitely diagnosed by polymerase chain reaction (PCR) tests from the transbronchial biopsy (TBB) specimen and followed by surgery.

## Case presentation

**Fig. 1:**
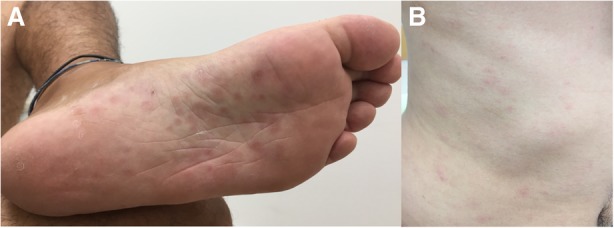
Erythematous popular rash. **a**: Erythematous popular rash over the solesm, **b**: Erythematous popular rash over the abdomen

**Fig. 2:**
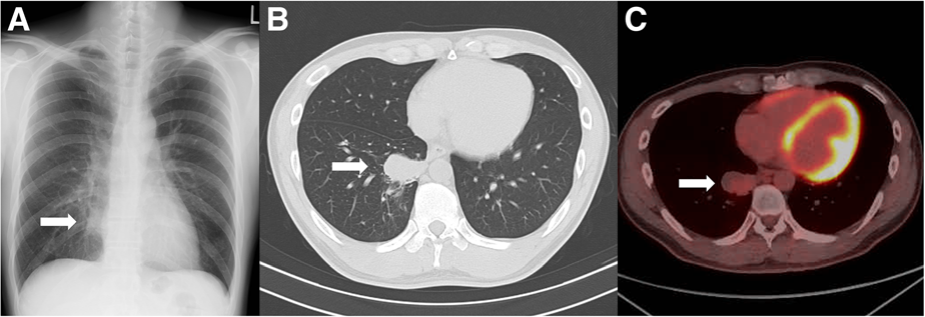
Images of the lung mass. **a** Chest X-ray on the first visit to our hospital. A mass lesion was shown in the right lower field (arrow), **b** Chest computed tomography on the first visit to our hospital. A single mass lesion (4 cm in size) was shown in the right lower lobe of the lung (arrow), **c** Fluorodeoxyglucose-positron emission tomography (FDG-PET) before the surgery, 4 months after the first visit. A single mass lesion was still remained in spite of the antibiotic treatment. It had abnormal uptake with a maximal standardized uptake value (SUV max) of 2.51 (arrow)

A 34-year-old Japanese heterosexual man presented to our hospital with a 4 cm heterogeneous mass in the right lower lobe (Fig. 2). He had had a symptom of productive coughing, sore throat and nasal discharge for 5 days, but he had no fever and no dyspnea, and his general condition was good. He had a medical history of minimal lesion nephrotic syndrome and had received corticosteroid therapy until 4 months prior to his first visit to our institution. He was a current smoker (15 pack-years). He had had sexual intercourse with a woman other than his wife 4 months prior to his first visit. Physical examination revealed right inguinal nontender enlarged lymph nodes, and erythematous papular rash over the palms, soles and abdomen (Fig. 1). However, cervical and supraclavicular lymph nodes were not palpable, and he did not have abnormal neurologic findings.

C-reactive protein level was elevated at 1.02 mg/dL as shown in the laboratory tests (Table 1). The rapid plasma reagin (RPR) and *Treponema pallidum* hemagglutination test (TPHA) revealed titers 1:64 and 1:5,120, respectively, although Human immunodeficiency virus testing was negative. Chest X-ray (Fig. 2a) and computed tomography (Fig. 2b) revealed a single mass lesion (4 cm in size) in the right lower lobe, and enlarged lymph nodes (4.5 cm in size) in the right inguinal region.

**Table 1:** Laboratory findings on the first visit to our institution

< Blood cell count >	
White blood cell	7,150 /μL
Red blood cell	520 × 10^4^ /μL
Hemoglobin	14.8 g/dL
Platelet	27.8 × 10^4^ /μL
< Serum chemistry>	
Total protein	8.1 g/dL
Albumin	4.6 g/dL
Total-bilirubin	0.5 mg/dL
Alkaline phosphatase	252 IU/L
Aspartate transaminase	15 IU/L
Alanine transaminase	23 IU/L
γ-Glutamyl transpeptidase	30 IU/L
Lactate dehydrogenase	158 IU/L
Blood urea nitrogen	11 mg/dL
Creatinine	0.84 mg/dL
C-reactive protein	1.02 mg/dL
Sodium	141 mmol/L
Potassium	4.4 mmol/L
Chlorine	103 mmol/L
< Coagulation>	
Prothrombin time (International normalized ratio)	1.09
Activated partial thromboplastin time	50 s
< Infection >	
Rapid plasma reagin test	Positive (titers 1:64)
*Treponema pallidum* hemagglutination test	Positive (titers 1:5,120)
Hepatitis B surface antigen	Negative
Hepatitis C antibody	Negative
Human immunodeficiency virus antibody	Negative
Aspergillus antigen	Negative
Cryptococcus antigen	Negative
< Tumor marker >	
Carcinoembryonic antigen	< 1 ng/mL
Soluble cytokeratin fragment	0.5 ng/mL
Pro-gastrin releasing peptide	27.0 pg/mL
<Autoantibody>	
Proteinase3-antineutrophil cytoplasmic antibody	< 1 U/mL
Myeroperoxidase-antineutrophil cytoplasmic antibody	< 1 U/mL

**Fig. 3:**
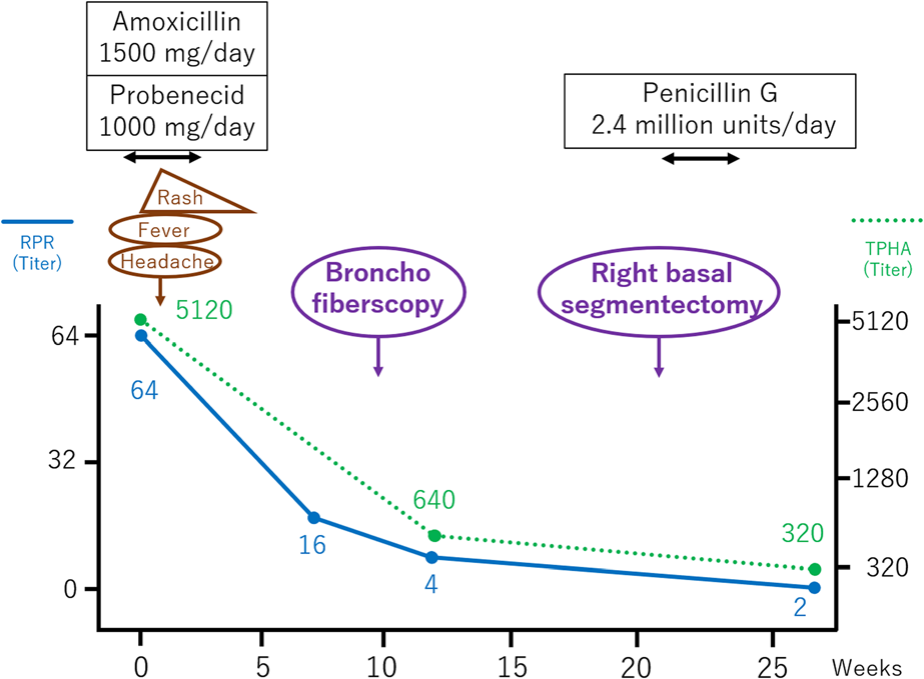
Clinical course of the treatment. The induction of the oral antibiotics caused fever, headache and exacerbation of erythematous papular rash on the next day, which was considered as Jarisch-Herxheimer reaction. Treatment for 2 weeks improved the rash and serological data. However, the lung mass had not changed in size. Surgical resection was followed, and then, additional intravenous antibiotics for 2 weeks was administered. Abbreviation; rapid plasma reagin test: RPR; *Treponema pallidum* hemagglutination test: TPHA

**Fig. 4:**
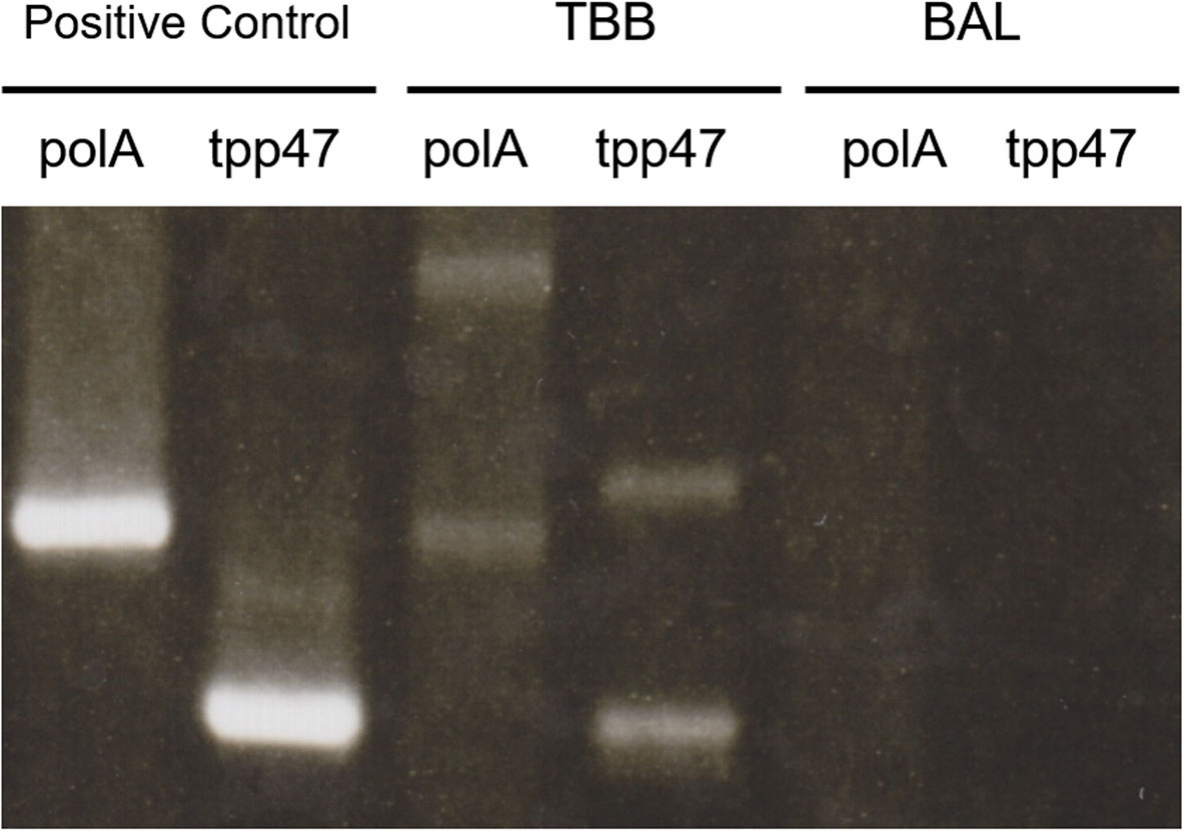
Electrophoresis of the amplified products from the lung mass with polymerase chain reaction (PCR) techniques. BAL was performed with 20 mL saline. The two types of gene fragments of *Treponema pallidum* (tpp47 and polA) acquired from bronchoalveolar lavage (BAL) and transbronchial biopsy (TBB) was amplified by PCR techniques. Both gene fragments were positive in samples from TBB, but not BAL

**Fig. 5:**
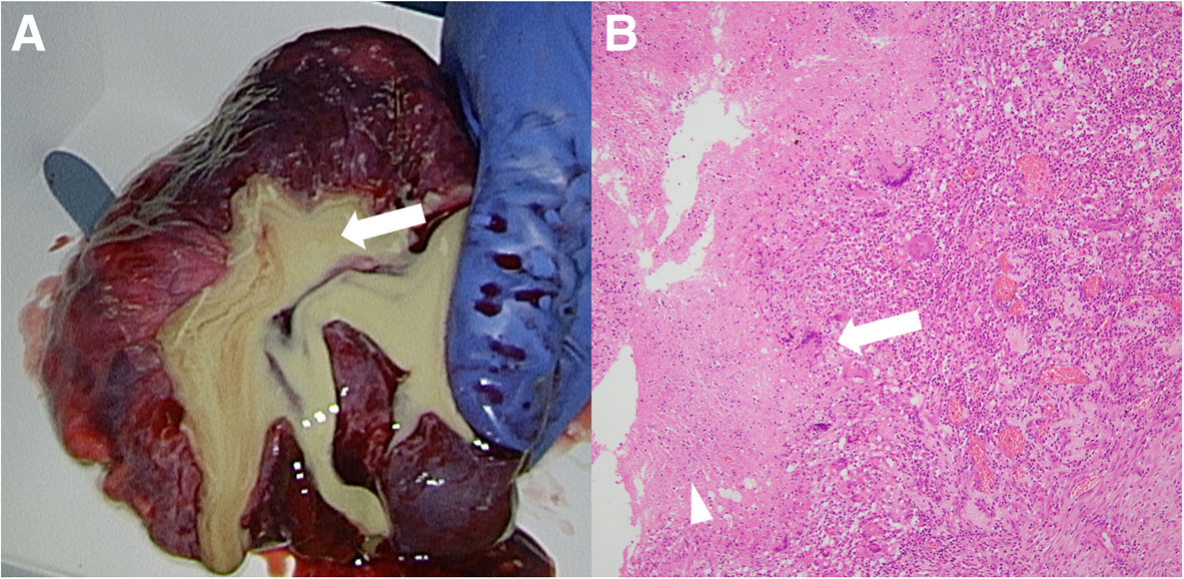
Gross and microscopic pathology of lung specimens obtained by surgery **a** Gross pathology showed pus inside the lung abscess (arrow), **b** Microscopic pathology showed granuloma formation by epithelioid histiocytes and Langhans giant cells (arrow), in addition to necrosis (arrow-head). Original Magnification X100. Hematoxylin and eosin (HE) staining

Diagnosed as secondary syphilis, amoxycillin 1500 mg per day and probenecid 1000 mg per day were orally administered for 2 weeks. Subsequently, rash, inguinal lymph nodes and serological markers were improved (Fig. 3), however, the lung mass remained unchanged in size (Fig. 2c). TBB confirmed the pulmonary involvement of syphilis by PCR techniques (tpp47-, and polA-PCR) (Fig. 4), whereas malignancy and other possible infections such as bacteria and fungi were negative (Table 2). Five months after the first visit, right basal segmentectomy was performed to exclude other comorbid diseases, especially malignancy. The remained lung mass was an abscess and histological analysis showed the granuloma formation by epithelioid histiocytes and Langhans giant cells with necrosis (Fig. 5). The comprehensive PCR tests for multi-microbes were performed in the resected lung specimens, and no microbes were significantly positive (Table 2). Subsequently, penicillin G 2.4 million units per day was intravenously administered for 2 weeks, and the pulmonary involvement has resolved without relapse after 8 months follow-up.

**Table 2:** Microbiological analysis in specimens obtained by bronchofiberscopy and surgery

1. Bronchofiberscopy		
PCR tests for *Treponema pallidum*		
Bronchoalveolar lavage		Undetected
TBB		Detected (tpp47-PCR and polA-PCR)
Culture tests for bacteria and mycobacteria		
Bronchoalveolar lavage		Undetected
Lavage of forceps in TBB		Undetected
2. Surgery		
Real-time PCR tests for *Treponema pallidum*		Undetected
Culture test for bacteria in pus inside the abscess		Undetected
Real-time PCR tests for multi-microbes [17]		
Number	Bacteria name	Quantity
1	*Staphylococcus aureus*	Undetected
2	*Bacillus anthracis*	Undetected
3	*Listeria monocytogenes*	Undetected
4	*Streptococcus pyogenes*	Undetected
5	*Streptococcus agalactiae*	Undetected
6	*Streptococcus mutans*	Undetected
7	*Streptococcus sobrinus*	Undetected
8	*Streptococcus sanguinis*	Undetected
9	*Streptococcus oralis*	Undetected
10	*Streptococcus salivaris*	Undetected
11	*Streptococcus pneumoniae*	Undetected
12	*Enterococcus faecalis*	Undetected
13	*Enterococcus faecium*	Undetected
14	*Clostridium tetani*	Undetected
15	*Clostridium difficile*	Undetected
16	*Peptostreptococcus anaerobius*	Undetected
17	*Actinomyces*	Undetected
18	*Corynebacterium diphtheriae*	Undetected
19	*Mycobacterium tuberculosis*	Undetected
20	*Mycobacterium laprae*	Undetected
21	*Mycobacterium chelonae*	Undetected
22	*Mycobacterium kansasii*	Undetected
23	*Mycobacterium avium complex*	Undetected
24	*Nocardia asteroids*	Undetected
25	*Bacteroides fragills*	Undetected
26	*Elizabethkingia meningosepticum*	Undetected
27	*Campylobacter jejuni*	Undetected
28	*Helicobacter cinaedi*	Undetected
29	*Helicobacter pylori*	Undetected
30	*Rickettsia prowazekii*	Undetected
31	*Rickettsia japonica*	Undetected
32	*Orientia tsutsugamushi*	Undetected
33	*Bartonella henselae*	Undetected
34	*Brucella*	Undetected
35	*Bordetella pertussis*	Undetected
36	*Burkhoderia mallei*	Undetected
37	*Burkhoderia cepacian*	Undetected
38	*Neisseria gonorrhoeae*	Undetected
39	*Neisseria meningitidis*	Undetected
40	*Francisella tularensis*	Undetected
41	*Legionella pneumophilia*	Undetected
42	*Moraxella catarrhalis*	Undetected
43	*Pseudomonas aeruginosa*	Undetected
44	*Acinetobacter baumannii*	Undetected
45	*Aeromonas hydrophia*	Undetected
46	*Vibrio cholerae*	Undetected
47	*Vibrio parahaemolyticus*	Undetected
48	*Vibrio vulnificus*	Undetected
49	*Haemophilus influenzae*	Undetected
50	*Escherichia coli*	Undetected
51	*Salmonella enterica*	Undetected
52	*Shigella*	Undetected
53	*Klebsiella pneumonia*	Undetected
54	*Yersinia psttis*	Undetected
55	*Yersinia enterocolitica*	Undetected
56	*Citrobacter freundii*	Undetected
57	*Proteus mirabilis*	Undetected
58	*Morganella morganii*	Undetected
59	*Providencia*	Undetected
60	*Mycoplasma pneumoniae*	Undetected
61	*Fusobacterium nucleatum*	Undetected
62	*Leptospira interrogans*	Undetected
63	*Chlamydia psittaci*	Undetected
64	*Chlamydia trachomatis*	Undetected
65	*Chlamydia pneumoniae*	Undetected
66	*Aspergillus fumigatus*	Undetected
67	*Aspergillus nigar*	Undetected
68	*Aspergillus flavus*	Undetected
69	*Cryptococcus*	Undetected
70	*Histoplasma*	Undetected
71	*Trichosporon*	Undetected
72	*Mucor*	Undetected
73	*Coccidioides*	Undetected
74	*Propionibacterium acnes*	Detected (not significant)
75	*Stenotrophomonas maltophilia*	Detected (not significant)
76	*Candida albicans*	Detected (not significant)

*Abbreviations*: *TBB* Transbronchial biopsy, *PCR* Polymerase chain reaction

## Discussion and conclusions

This is a rare case of a lung abscess caused by secondary syphilis, that was diagnosed by PCR techniques in TBB. The abscess was not improved by antibiotics and required surgery.

Coleman showed the criteria for the clinical diagnosis of secondary syphilis with pulmonary involvement in 1983 [5], and several dozen cases have been reported [6–16]. In some of them, PCR was used for the diagnosis of pulmonary involvement (Table 3) [13–16]. PCR is useful for the diagnosis of the infection of *Treponema pallidum* [18, 19], because it is difficult to directly visualize *Treponema pallidum*. In those reports, PCR was used in samples from TBB, bronchoalveolar lavage (BAL), bronchial aspirate, or computed tomography-guided percutaneous needle aspiration (CTNA) [13–16]. Thus far, only one case has been reported on lung abscess caused by secondary syphilis, that was diagnosed by PCR in CTNA [15]. In our case, the results of PCR in samples from TBB, but not BAL, was positive. For the detection of some infectious diseases, TBB or the combination of BAL and TBB was reported to be useful [20, 21]. Thus, it could be important to perform TBB to detect the pulmonary involvement by *Treponema pallidum*.

**Table 3:** Reported cases of secondary syphilis with pulmonary involvement which was diagnosed by PCR techniques

Case no.	Age	Gender	Respiratory symptoms	Extrapulmonary symptoms	Chest imaging	Sample collection method	Reporting year	Reference
1	34	Male	Chest pain	Progressive weakening, anorexia, weight loss, and night sweats	Several bilateral, round, excavated opacities and subtracheal adenopathy	BAL	2006	[13]
2	49	Female	Dry cough	Disabling cervical pain, fever, and night sweats	Lung lobe parenchymal lesion	BAL and bronchial aspirate	2015	[14]
3	30	Male	Hemoptysis, chest pain, dyspnea	Fever and rash	a 3 cm, irregularly-shaped, well-defined consolidation and a 1 cm hilar node	CTNA	2018	[15]
4	62	Male	No respiratory symptoms	epigastric pain	Multiple nodular bibasilar subpleural nodules	TBB	2018	[16]

*Abbreviation*: *PCR* Polymerase chain reaction, *BAL* Bronchoalveolar lavage, *CTNA* Computed tomography-guided percutaneous needle aspiration, *TBB* Transbronchial biopsy

The lung abscess was not improved by 2 weeks of oral antibiotics. It may be because penetration of antibiotics into the abscess was impaired. We treated the present case with amoxicillin and probenecid, because there is no insurance coverage for intramuscular penicillin for syphilis in Japan. Administration of intravenous penicillin G was considered as a more potent antibiotic treatment. However, as in this case, it is necessary to consider surgical resection as the treatment for uncontrolled infection and in order to exclude other diseases, including malignancy, when the lung involvement is poorly improved by antibiotics.

Lung lesions associated with syphilis are still rare, but the reported cases have been increasing as the number of patients with syphilis increases [5–16]. Thus, we should consider chest X-ray in the cases of the patients with syphilis who have pulmonary symptoms.

In conclusion, to our knowledge, this is the first surgically treated case of a lung abscess caused by syphilis, which was diagnosed by PCR techniques in TBB. This report could propose a useful diagnostic method for the pulmonary involvement of syphilis.

## Data Availability

Not applicable.

## Abbreviations

BAL: Bronchoalveolar lavage
CTNA: Computed tomography-guided percutaneous needle aspiration
PCR: Polymerase chain reaction
RPR: Rapid plasma reagin test
TBB: Transbronchial biopsy
TPHA: *Treponema pallidum* hemagglutination test
